# FibroScan grading and metabolic correlates of fatty liver disease

**DOI:** 10.6026/973206300221487

**Published:** 2026-03-31

**Authors:** Hemant Kumar Jain, Jitendra Kanjolia, Aparna Mittal, Shivani Parihar

**Affiliations:** 1 Department of General Medicine, Government Medical College, Datia, Madhya Pradesh, India; 2 Department of Pathology, Chirayu Medical College, Bhopal, Madhya Pradesh, India

**Keywords:** Fatty liver disease, FibroScan, controlled attenuation parameter, metabolic syndrome, insulin resistance, liver fibrosis

## Abstract

Fatty liver disease is increasingly recognized as a hepatic manifestation of systemic metabolic dysfunction. This cross-sectional study, conducted among 165 adult patients with ultrasonographic evidence of fatty liver disease, evaluated metabolic risk factors and liver involvement using FibroScan for steatosis grading and liver stiffness measurement. The results revealed a high prevalence of metabolic syndrome, insulin resistance, dysglycemia and atherogenic dyslipidemia among participants, with moderate to severe hepatic steatosis being common. FibroScan grades showed a significant association between higher steatosis levels and worsening metabolic profiles, including body composition, glycemic parameters and insulin resistance. Thus, we show FibroScan as a valuable non-invasive tool for identifying patients at increased metabolic and hepatic risk, allowing for early risk stratification and management.

## Background:

Fatty liver disease, encompassing a spectrum of hepatic disorders characterized by excessive triglyceride accumulation within hepatocytes, represents a major and rapidly growing public health concern globally. It is increasingly recognized that hepatic steatosis is not an isolated liver condition, but rather the hepatic manifestation of systemic metabolic dysfunction involving obesity, insulin resistance, dyslipidemia and hypertension. Metabolic dysfunction-associated steatotic liver disease (MASLD), previously termed non- alcoholic fatty liver disease (NAFLD), is defined by the presence of hepatic steatosis in conjunction with one or more cardiometabolic risk factors in the absence of significant alcohol intake or other causes of liver disease [1, 2]. The prevalence of fatty liver disease is rising worldwide in parallel with the global epidemics of obesity and metabolic syndrome, with estimates indicating that MASLD affects a substantial proportion of the adult population and is a leading cause of chronic liver disease in both community and clinical settings [1, 3]. Epidemiological studies have consistently shown that individuals with metabolic syndrome have a significantly higher risk of developing both hepatic steatosis and fibrosis compared with those without metabolic abnormalities, highlighting the intertwined pathophysiology between systemic metabolic dysregulation and liver disease progression [4, 5]. Transient elastography with controlled attenuation parameter (CAP) and liver stiffness measurement (LSM) has emerged as a validated non-invasive modality for quantifying hepatic steatosis and fibrosis, respectively. CAP has demonstrated reasonable diagnostic accuracy for identifying and grading hepatic steatosis in patients at risk for MASLD and elevated CAP values are associated with key metabolic abnormalities including central obesity, hypertriglyceridemia and insulin resistance [6, 7]. Moreover, higher CAP and LSM values correlate with increasing numbers of metabolic syndrome components, reinforcing the clinical utility of these measures as indicators of systemic metabolic burden as well as hepatic involvement [8]. Given the high metabolic risk profile of patients with fatty liver disease, there is a need to systematically evaluate the relationship between elastography-derived steatosis and fibrosis grades and metabolic correlates. Understanding these associations can facilitate early risk stratification and comprehensive management of individuals with MASLD.

## Methodology:

This cross-sectional observational study was conducted at Dr. Hemant Jain's clinic, Vidhya Vihar colony, Datia, Madhya Pradesh, to evaluate liver stiffness and steatosis grading using transient elastography and analyze their association with metabolic parameters in individuals diagnosed with fatty liver disease. Adult patients attending outpatient and inpatient services, suspected to have fatty liver disease based on clinical evaluation and/or ultrasonographic evidence of hepatic steatosis, were screened for eligibility. Inclusion criteria included patients aged ≥18 years with ultrasonographic evidence of fatty liver and the ability to provide informed written consent, while exclusion criteria included significant alcohol consumption, known chronic liver diseases, use of hepatotoxic drugs, decompensated liver disease, pregnancy and others. Sample size calculation, based on an anticipated 50% prevalence of metabolic abnormalities, estimated a minimum of 150 participants and 175 participants were enrolled, with complete data obtained from 165 participants. Data collection involved demographic, clinical and anthropometric measurements, followed by biochemical analysis of fasting plasma glucose, glycated hemoglobin (HbA1c), serum insulin, lipid profile and liver function tests. FibroScan® (Echosens, Paris, France) was used for transient elastography and liver stiffness measurement (LSM) and controlled attenuation parameter (CAP) values were recorded to grade steatosis and assess liver fibrosis. Steatosis grades were categorized as S0 (no steatosis) to S3 (severe steatosis) and metabolic syndrome was defined according to standard criteria. Statistical analysis was performed using standard software, with correlations between FibroScan-derived parameters and metabolic variables assessed using correlation coefficients and a p-value < 0.05 considered statistically significant.

## Results:

A total of 165 participants completed the study and were included in the final analysis. The study population predominantly comprised middle-aged adults, with a higher proportion of males and exhibited a substantial burden of adiposity and cardiometabolic risk factors, indicating a metabolically vulnerable cohort (Table 1 - see PDF). The biochemical profile demonstrated a high prevalence of dysglycemia, insulin resistance and atherogenic dyslipidemia, collectively supporting the close metabolic association of fatty liver disease within this population. Liver enzyme elevations were modest overall, reinforcing the limited sensitivity of transaminases in reflecting disease severity in fatty liver states (Table 2 - see PDF). FibroScan-based assessment revealed that the majority of participants had moderate to severe hepatic steatosis, with only a small subset showing minimal or no steatosis. This distribution underscores the tendency for patients to present at more advanced steatosis stages by the time of clinical evaluation (Table 3 - see PDF). In parallel, nearly half of the cohort demonstrated evidence of at least significant fibrosis, highlighting the silent progression of fibrotic changes in fatty liver disease (Table 4 - see PDF). A clear and graded relationship was observed between increasing steatosis severity and worsening metabolic parameters. Higher steatosis grades were associated with greater adiposity, central obesity, impaired glycemic control, hypertriglyceridemia and escalating insulin resistance, suggesting a dose-response pattern between hepatic fat accumulation and systemic metabolic dysfunction (Table 5 - see PDF). The prevalence of metabolic syndrome increased markedly across steatosis grades, with the highest burden observed among individuals with severe steatosis. This finding reinforces the concept of fatty liver disease as a hepatic manifestation of systemic metabolic derangements rather than an isolated liver condition (Table 6 - see PDF).

## Discussion:

In this study of 165 patients with fatty liver disease, non-invasive assessment using FibroScan indicated that higher controlled attenuation parameter (CAP) values and liver stiffness measurements (LSM) were closely associated with adverse metabolic profiles. Our observation of a graded increase in metabolic abnormalities with higher steatosis grades reinforces findings from previous cross-sectional studies showing that CAP and LSM correlate with the number and severity of metabolic syndrome components such as obesity, dysglycemia and insulin resistance [9]. These associations support the use of transient elastography not only for liver grading but also as an integrated marker of systemic metabolic dysfunction in fatty liver disease. Several studies have underscored that patients with more components of the metabolic syndrome are more likely to have both higher CAP and LSM values, indicating that metabolic derangements and fibrotic progression occur concomitantly in at-risk cohorts [10]. This pattern parallels the increasing prevalence of metabolic syndrome components observed across steatosis grades in our study and suggests a shared pathophysiological trajectory between hepatic fat accumulation and broader cardiometabolic risk. Beyond metabolic characterization, the prognostic implications of transient elastography parameters have been investigated extensively in longitudinal cohorts.

For example, elevated liver stiffness has been consistently linked to a higher risk of liver-related clinical events in patients with metabolic dysfunction-associated steatotic liver disease, emphasizing its utility in risk stratification beyond cross-sectional assessment [11]. Moreover, in population-based analyses, hepatic fibrosis defined by elastography was associated with multiple cardiometabolic risk factors, including obesity, diabetes, hypertension and low HDL cholesterol, indicating that fibrotic advancement reflects systemic cardiometabolic burden [12]. While most research focuses on liver- related outcomes and metabolic correlations, there is emerging evidence that steatosis and fibrosis assessed by transient elastography may also relate to extrahepatic conditions, such as chronic kidney disease, particularly in metabolic disease cohorts [13]. These broader associations highlight the systemic nature of steatotic liver disease and the importance of comprehensive evaluation. Overall, the current findings support a model in which increasing steatosis and fibrosis-as measured by CAP and LSM-are integrated markers of metabolic dysfunction and may carry prognostic significance for both hepatic and extrahepatic disease burden.

## Conclusion:

We show that FibroScan-based assessment of hepatic steatosis and fibrosis provides meaningful insight into the metabolic severity of fatty liver disease. Increasing steatosis grades were closely associated with adverse metabolic correlates and a higher burden of fibrosis, underscoring the systemic nature of the disease process. Thus, we show the role of transient elastography as a practical, non-invasive modality for identifying patients at greater cardiometabolic and hepatic risk, enabling earlier stratification and targeted intervention in routine clinical practice.
